# Whey Proteins Isolate-Based Biopolymeric Combinations to Microencapsulate Supercritical Fluid Extracted Oleoresins from Sea Buckthorn Pomace

**DOI:** 10.3390/ph14121217

**Published:** 2021-11-24

**Authors:** Liliana Mihalcea, Iuliana Aprodu, Loredana Dumitrașcu, Elena Iulia Cucolea, George-Mădălin Dănilă, Elena Enachi, Vasilica Barbu, Oana Emilia Constantin, Leontina Grigore-Gurgu, Nicoleta Stănciuc

**Affiliations:** 1 Faculty of Food Science and Engineering, Dunărea de Jos University of Galati, Domnească Street 111, 800201 Galati, Romania; Liliana.Gitin@ugal.ro (L.M.); Iuliana.Aprodu@ugal.ro (I.A.); Loredana.Dumitrascu@ugal.ro (L.D.); Elena.Ionita@ugal.ro (E.E.); Vasilica.Barbu@ugal.ro (V.B.); Emilia.Constantin@ugal.ro (O.E.C.); Leontina.Gurgu@ugal.ro (L.G.-G.); 2 Cromatec Plus SRL, Research Center for Instrumental Analysis SCIENT, Petre Ispirescu Street 1, 077176 Tâncăbești, Romania; iulia.cucolea@scient.ro (E.I.C.); george.danila@scient.ro (G.-M.D.)

**Keywords:** sea buckthorn, supercritical extraction, oleoresin, microencapsulation, Maillard, antibiabetic, antioxidant

## Abstract

In this study, high-value, carotenoid-rich oleoresin obtained by supercritical carbon dioxide (SFE-CO_2_) extraction was used to develop five variants of microencapsulated delivery system, based on whey proteins isolate (WPI), in combination with inulin (I), pectin (P) or lactose (L). The WPI:I and WPI:L variants were also obtained by conjugation via Maillard reaction. The microencapsulation of the SFE-CO_2_ sea buckthorn pomace oleoresin was performed by emulsion, complex coacervation and freeze-drying, which allowed for the obtaining of five powders, with different phytochemicals profile. The WPI:I conjugate showed the highest level of total carotenoids, whereas the counterpart WPI:L showed the highest content in linoleic acid (46 ± 1 mg/g) and palmitoleic acid (20.0 ± 0.5 mg/g). The β-tocopherol and β-sitosterol were identified in all variants, with the highest content in the conjugated WPI:L variant. Both WPI:L and WPI:I conjugate samples presented similar IC50 value for inhibitory activity against pancreatic lipase and α-amylase; the highest activity was observed for the conjugated WPI:I. The WPI:P combination allowed the highest release of carotenoids in the gastro-intestinal environment. All the powders exhibited poor flowing properties, whereas water activity (a_w_) ranged from 0.084 ± 0.03 to 0.241 ± 0.003, suggesting that all variants are stable during storage. In case of solubility, significant differences were noticed between non-heated and glycated samples, with the highest value for the WPI:I and the lowest for glycated WPI:I. The structural analysis revealed the presence of finer spherosomes in WPI:I and WPI:L, with a reduced clustering capacity, whereas the particles in the conjugated samples were more uniform and aggregated into a three-dimensional network.

## 1. Introduction

Nowadays, the use of natural pigments in food, pharmaceutical and cosmeceutical applications or as nutraceuticals, has emerged due to a wide biological impact, associated with free radical scavenging, antimicrobial activities, prevention of carcinogenesis, radioprotection, anti-stress, regulating immunity, preventing cardiovascular disease and tissue regeneration abilities, etc. [1,2]. Additionally, these bioactives are found in various foods, especially vegetables and fruits, commonly consumed by the population, without health risks [3].

In this regard, sea buckthorn (*Hippophaë rhamnoides* L.) holds a special attention due to a comprehensive nutritional and health-promoting properties, including reduction of fever, inflammation, antitoxic effect, a positive effect on the regeneration and condition of the skin and hair [4,5]. The positive health-associated benefits were scientifically demonstrated, such as antioxidant, cardioprotective, hypoglycemic, hypolipidemic, and antibacterial [6]. For example, sea buckthorn berries are known as a rich source of nutrients and bioactives, including vitamins, carotenoids (β-carotene, zeaxanthin, lutein, lycopene, etc.), polyphenols, flavonoids, organic acids, pectin, carbohydrates, polyunsaturated fatty acids and essential amino acids [7]. In addition, Dąbrowski et al. [8] suggested that sea buckthorn could accumulate into two distinct oils, different in composition, as one is derived from seeds, and the other oil can be found in fruit flesh. The later includes extremely high contents of ω-7 palmitoleic acid and carotenoids, which are rarely found in nature. The health benefits associated with palmitoleic acid administration are related to the immune-metabolic effects in adipose tissue [9], diminishing the effect of macrophage activation and skeletal muscle insulin resistance [10], increasing insulin sensitivity and, therefore, reducing the risk of diabetes [11]. In addition, palmitoleic acid may induce beneficial adipocyte-derived lipid hormone (lipokine) released to prevent the harmful effects of adiposity and excess non-esterified fatty acids on systemic glucose metabolism [12].

Reported by Tkacz et al. [5] as a multi-purpose plant, industrial processing of sea buckthorn (SB) includes different food products (juice, drink, smoothie, jam, sauce, oil) or alcohols (wine, liqueur, beer additive) from berries. Berries are not only valuable, but also, herbal leaf teas may be used as sources of flavonoids for its detoxifying properties, production of fodder supplements of sea buckthorn by-products, cosmetics, pharmaceuticals, and fuel as firewood. However, when processing, a significant amount of pomace, discarded as waste, a high pollution potential is obtained. The sea buckthorn pomace (SBP), consisting of skin and seeds, may be considered a valuable source of flavonoids, carotenoids, phenolic compounds, polyunsaturated fatty acids, and vitamins, with multiple functions. This in turn, raises the issue of the bioactives extraction and stabilization, from the perspective of facilitating their various applications and controlled release. In general, the bioactives stability and release is affected by environmental conditions, including alkali, oxygen, enzymatic activities, ultraviolet, metal ions, and different pH conditions in the digestive tract, causing degradation and hence decreasing their stability and bio-accessibility upon ingestion. Therefore, microencapsulation is considered a supporting technology for protecting these bioactive compounds for use in food, pharmaceuticals, nutraceuticals and cosmeceuticals, allowing a controlled release in the gastrointestinal environment. Different techniques were reported for SB bioactives encapsulation, such as: complex coacervation and freeze-drying [13,14] or nano-emulsions fabricated by ultra-high pressure homogenization process [15]. For example, Chang et al. [15] prepared SB pulp oil nano-emulsions based on caseinate and whey proteins by ultra-high pressure homogenization to improve the poor water solubility and instability of the oil. These authors suggested that both nano-emulsions had good microstructures and rheological properties, being promising carriers for extending the applications of SB pulp oil as nutraceutical or functional dairy beverage. In our previous study, the cross-linked reaction mediated by transglutaminase was used to improve the phytochemical and physico-chemical properties of the SB microencapsulated oils [14]. 

The aim of this study was to investigate different combinations of biopolymers for encapsulating the sea buckthorn pomace (SBP) oleoresins obtained using the CO_2_ supercritical fluid extraction (SFE-CO_2_) method. Whey protein isolate (WPI) -based biopolymer combinations were used with inulin, pectin and lactose via emulsion, complex coacervation and freeze-drying. A comparative analysis on the ability of structural changes of WPI conjugated with inulin (I) and lactose (L) through Maillard wet-heating reaction on entrapping efficiency and the properties of the related powders was also tested. The SBP oleoresins and powders were characterized for phytochemical content (total carotenoids, individual carotenoids, fatty acids, phytosterols, and tocopherols) and antioxidant activity. The free extract and microcapsules were analyzed before and after simulated digestion for carotenoids, whereas the powders were analysed for inhibitory effects on enzymes associated with the metabolic syndrome. The structural particularities of the powders were analysed using laser confocal microscopy. The powders were also analyzed for physical characteristics, such as bulk density, tapped density, Carr Index, Hausner ratio, water activity and solubility. 

## 2. Results and Discussion

### 2.1. Advanced Phytochemical Characterizations of the Extract and Powders

A comparison of phytochemical profile and antioxidant activity of the SBP oleoresin obtained by SFE-CO_2_ and microcapsule powders is shown in Table 1. As expected, the SFE-CO_2_ oleoresins from SBP presented higher levels of phytochemicals. In relation to total carotenoids, the CO_2_-SFE oleoresins from SBP showed a content of 510 ± 8 mg/g DW, whereas the powders showed variable content, from 120.0 ± 0.6 mg/g DW in V2 to 199.0 ± 0.4 mg/g DW in V4. From Table 1 it can be observed that the cross-linking process of the biopolymeric materials in V4 and V5 due to heating allowed a higher retention of carotenoids, in terms of total carotenoids, β-carotene and lycopene. Although WPI are known for their good emulsifying properties, in this study it seems that conjugation of WPI with I and L allowed for a better microencapsulation of SBP oleoresin, due to a combination of the emulsifying capacities of proteins with the solvating characteristics of polysaccharide [16]. From this point of view, due to the enhanced emulsifying properties of the protein-polysaccharide conjugates, the oleoresins retentions in variants with Maillard-based induced reaction were higher. This phenomenon occurs due to their amphiphilic nature, in which the protein hydrophobic groups are able to adsorb the lipid phase, and the polysaccharide chains (which are strongly hydrophilic) can easily solvate the aqueous medium [17]. Therefore, all the eleven fatty acids identified in the SBP oleoresin obtained by SFE-CO_2_ were preserved, to different extents, in all the powders. 

From Table 1, it can be observed that the fatty acids profile revealed the presence of linoleic acid (C18:2) as a predominant in the extract (158 ± 1 mg/g), whereas different concentrations were found in microcapsule powders. The maximum retention of linoleic acid (C18:2) was found in V5, suggesting the hypothesis that the Maillard-based conjugates between WPI and L induced by heating in alkali conditions, facilitated the entrapment of a higher amount of 46 ± 1 mg/g. This trend was observed for all fatty acids (Table 1), with a particular emphasis on the palmitoleic acid (C16:1) concentrations, which was found to range between 58 ± 1 mg/g in the extract to 8 ± 1 mg/g in V3 and a significant higher value in V5 of 20.1 ± 0.4 mg/g. The total content of fatty acids in the oleoresin extract was 523 mg, whereas in powders the smallest total content was found in V3 (74 mg) and the highest in V5 (165 mg).

The predominant tocopherols and phytosterols in the extract and, consequently in the powders, were β-tocopherol and β-sitosterol in the extract, whereas the powders showed a higher concentration in α-tocopherol, with the exception of V2. The Maillard-based conjugates between WPI and L obtained in V5 seems to favor the microencapsulation of bioactives from SPB oleoresin, due in particular to the enhanced ability of WPI conjugates to form a tighter interfacial layer structure with better entrapping ability. In both cases, the use of naturally occurring polysaccharides (I) and disaccharides (L) led to a higher antioxidant activity of the powders, thereby favoring the potential inhibition of lipid oxidation [18]. The highest antioxidant activities were found for V4 and V5, amounts of around 14 mMol/g DW.

The microencapsulation efficiency (ME) of total carotenoids varied between 87% in V3 and 91% in V2 (Table 2), being affected by the biopolymeric combinations. From Table 2, it can be observed that WPI:P (V2) combination yielded the highest ME for total carotenoids and β-carotene, whereas the highest ME for lycopene was found for V4, of 86%. Therefore, it can be appreciated that the type of biopolymeric combination used in microencapsulation highly influenced the microencapsulation efficiency and retention of the compounds into the microparticles core. 

### 2.2. In Vitro Digestion

**Figure 1:**
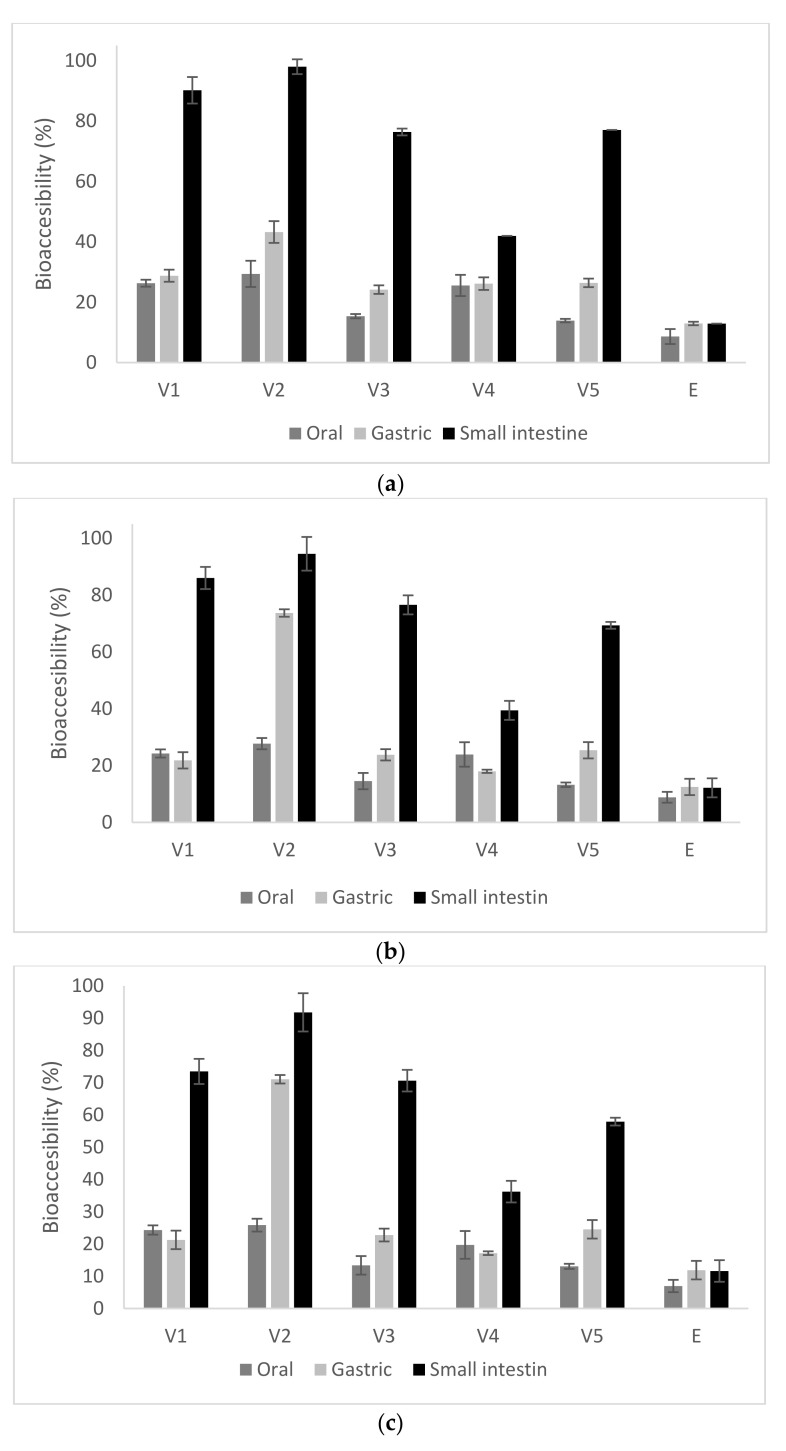
Bio-accessibility of total carotenoids (**a**), β-carotene (**b**) and lycopene (**c**) in in vitro digestion of microencapsulated variants (E – Extract, V1—whey proteins isolate:inulin, V2—whey proteins isolate: pectin, V3—whey proteins isolate:lactose, V4—whey proteins isolate:inulin in conjugated form, V5—whey proteins isolate:lactose in conjugated form).

In order to provide the health benefit in the human body, the carotenoids have to be absorbed first. Therefore, the SBP oleoresins and microcapsule powders were subjected to in vitro digestion in order to study the impact of microencapsulation on the bio-accessibility of the carotenoids. Bio-accessibility is defined as “the fraction of a compound that is released from its matrix in the gastrointestinal tract and thus becomes available for intestinal absorption” [19]. An in vitro static digestion simulated system was applied, including a short oral digestion phase, a gastric and small intestinal one, as described by Szabo et al. [20]. The total carotenoids, β-carotene and lycopene release (%) of the three phases is shown in Figure 1. 

From Figure 1, it can be observed that the carotenoid contents in the digestion supernatants changed significantly through three digestion phases. Therefore, significant differences were found among the five variants of microencapsulated samples during the digestion process in the mouth. The rate of total carotenoids released from the extract (8%) was significantly lower than that from the microcapsules (*p* < 0.05), where the release registered the lowest value in V3 (15.2 ± 0.2%) and the highest in V2 (29.1 ± 0.4%), suggesting that the microcapsules were more easily broken down under the oral simulated conditions, releasing the carotenoid to the systems. The release of the carotenoids in the first stage of digestion indicates that the oral phase highly contributes to the hydrolysis of carbohydrates from microencapsulation matrices; therefore, the release of carotenoids was favored due to their partial digestion. The total carotenoids values of five supernatants in the gastric phase was observed to have an increasing trend, from about 24% in V3 to approximatively 43% in V2, respectively. The higher release of carotenoids in the gastric digestion may be due to the fact that WPI molecules could be digested by proteases in the simulated gastric digestion process, which would also cause dissociation of the microcapsules [21]. Therefore, it seems that the WPI:P combination allowed for a higher release of carotenoids in the gastric environment, compared with the other variants. 

The disruption of microparticles in gastric simulated juice favored the release in the small intestinal juice, where the total carotenoids contents were found to increase progressively in all variants, significantly higher than similar values in the mouth stage, reaching a maximum value of approximatively 98% in V2. During the whole digestion process, the highest carotenoids content of the supernatant was observed in case of V2, followed by V1, V5 and V3, with the lowest content in V4 (Figure 1). When considering the in vitro digestion of the total carotenoids from the SPB oleoresins, the bio-accessibility reached significant lower values of approximatively 9% in oral digestion and about 13% in gastric and intestinal phases, respectively. The different behavior of the microencapsulated variants during in vitro digestion may be explained by the changes in electrostatic interactions between the polymers and enzymatic action. Therefore, the significant release of carotenoids during digestion may be due to the fact that the molecules are negatively charged at the neutral pH of the simulated saliva and small intestine [22]. Therefore, an electrostatic repulsion occurred between them, promoting the dissociation of the microcapsules can be considered.

The obtained results show that the microencapsulation of SBP oleoresins within protein-di- and -polysaccharides matrices by emulsion-complex coacervation and freeze-drying successfully improved the bio-accessibility of carotenoids. Gomez-Mascaraque et al. [23] reported that during in vitro digestion, the low pH of the gastric phase could induce the increased of cis-isomers β-carotene content, as a result of the pH driven isomerization of carotenoids into respective cis-isomers after their consumption.

### 2.3. Inhibitory Effect on Metabolic Syndrome-Associated Enzymes

Obesity, diabetes mellitus and related inflammation-related disorders are recognized as major health issues worldwide, with a significant increase in people suffering, which can lead to adverse chronic conditions such as cardiovascular diseases, certain cancers, hypertension and sleep-breathing disorders. Lipoxygenase, pancreatic lipase and α-amylase occur in the small intestine lumen, being responsible for oxygenation of the polyunsaturated fatty acids and the hydrolysis of carbohydrates and lipids, respectively. The inhibition of these enzymes will delay the absorption of monosaccharides and fatty acids, being an accepted alternative for the effective management of metabolic-syndrome-associated disorders in order to replace the conventional drugs and to develop a protective effect towards chronic non-communicable diseases, including type 2 diabetes, cardiovascular diseases and certain types of cancer [24]. It has been stated that the synthetic drugs produce several side effects, such as excessive lowering of blood sugar levels when used in combination with other medications meant to treat diabetes, abdominal pain, diarrhoea, and flatulence [25]. In our study, the potential of microencapsulated variants to inhibit selected enzymes involved in the metabolic syndrome, such as lipoxygenase, pancreatic lipase and α-amylase was tested. The inhibitory activities of the powders on the selected enzymes, involved in the metabolic syndrome-associated enzymes expressed as IC50 (µg/mL) are given in Table 3. 

The powders showed different IC50 values for LOX, with the highest value registered for V4 (37 ± 1 µg/mL, followed by V2 and V1. The inhibitory effect of the microcapsule powders on LOX may be described as: V4 > V2 > V1 > V3 > V5. The highest inhibitory effect on LOX activity of approximatively 83% was registered in case of V5. 

Regarding the in vitro inhibitory effect of microencapsulated variants on pancreatic lipase, the enzymes activity was inhibited in a dose-dependent manner, reaching a maximum of 96% for V5 at a concentration of 1 mg/mL. 

In case of α-amylase, V2 showed no inhibitory activity, whereas the highest value was obtained for V5, of 29.0 ± 0.3 µg/mL. The in vitro inhibitory effect on α-amylase also followed a dose-dependent trend, with the maximum inhibition at concentration of 1 mg/mL for all variants. However, the maximum inhibition effect of approximatively 83% was registered for V5, whereas the lowest of about 56% was found for V1. 

Condurache et al. [26] used several drugs as positive controls, such as acarbose, Orlistat and quercetin, and estimated IC50 values of around 4.5 µg/mL for α-amylase, 3 µg/mL for lipase and 2 µg/mL for LOX.

**Figure 2:**
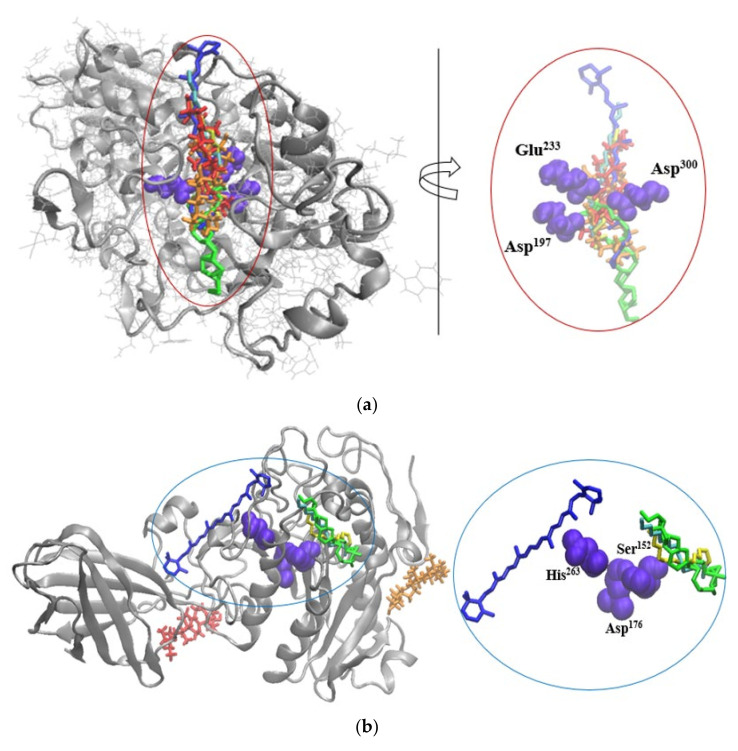

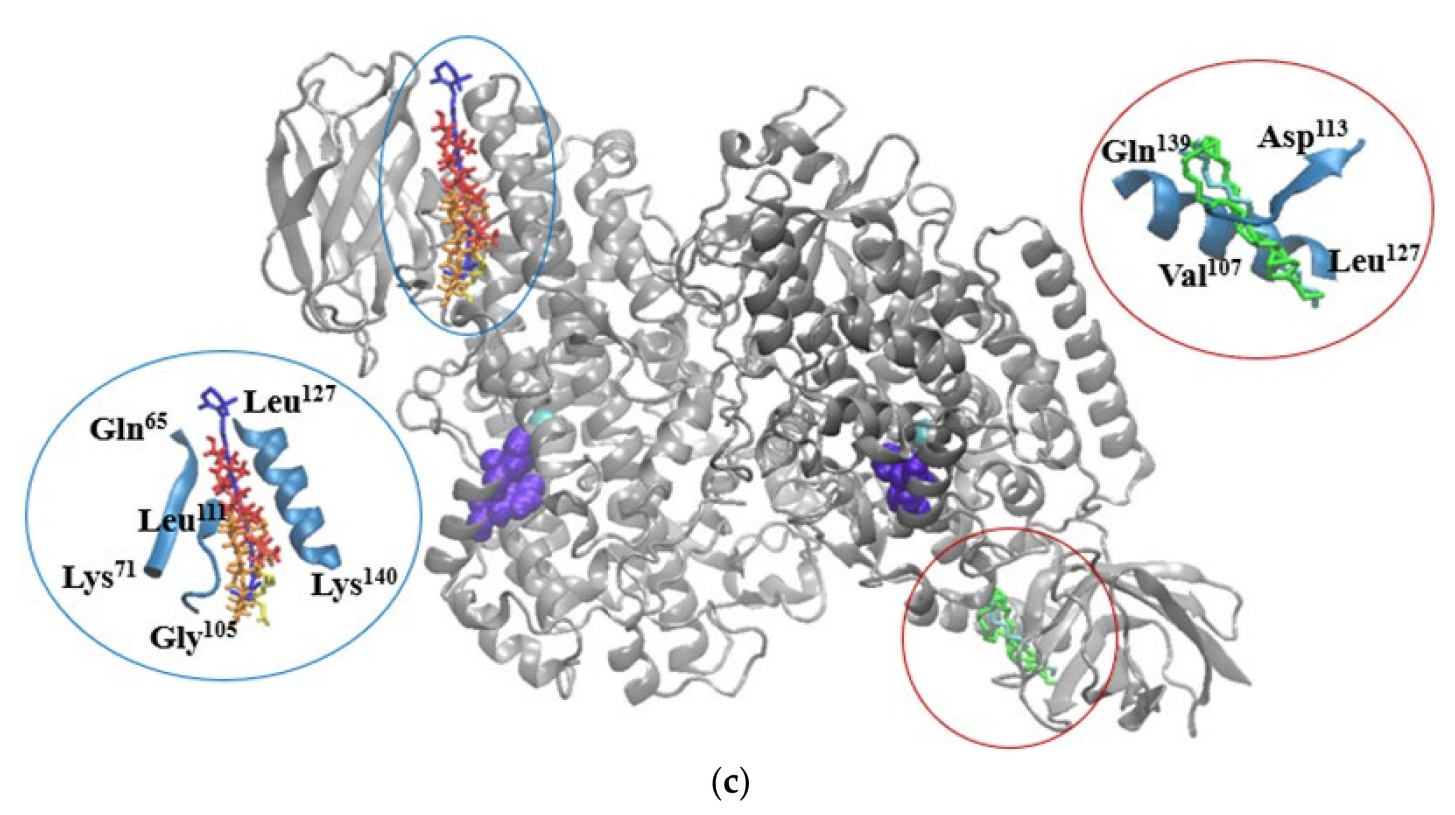
Superposition of the molecular docking results showing the complexes formed by α-amylase (**a**), lipase (**b**), and lipoxygenase (**c**), represented New Cartoon style in silver, with BCR (blue), BST (red), BTF (orange), LA (green), OA (cyan), and/or PA (yellow) represented in Licorice style. The catalytic amino acids establishing contacts with the ligands are represented in violet in Van der Waals style. In the insets details are given on the binding of the ligands to the catalytic amino acids of α-amylase (**a**), and lipase (**b**), or to the binding patches on lipoxygenase surface (**c**). Images were prepared using VMD software [30].

In the attempt to gather further understanding of the potential mechanism responsible for inhibiting the activity of these metabolic syndrome-associated enzymes, the molecular models of the complexes predicted through in silico docking tests were analyzed in-depth. As one can see in Figure 2a, all major phytochemicals quantified in the SBP oleoresins are prone to attach to the α-amylase surface, in the vicinity of the catalytic site. It appears that all tested ligands compete for interacting with Asp^197^, Asp^300^ and Glu^233^ amino acids, thereby, interfering with the specific catalytic activity of this triad [27].

In a similar manner, the BCR, LA, OA, and PA molecules appear to potentially affect the activity of lipase. As depicted in Figure 2b, BCR is in the close vicinity of His^263^, whereas LA, OA, and PA establish hydrophobic contacts with a second catalytic amino acid, namely Ser^152^ [28]. This kind of phytochemicals binding behavior might restrict or delay the substrate recognition and/or transformation by the enzyme. In case of lipoxygenase, none of the investigated ligands is able to attach with good affinity in the neighborhood of the active site or the catalytic iron. Therefore, there is no direct involvement of the major phytochemicals from SBP oleoresins in hampering the substrate recognition and there no direct competition with its binding to the specific site of lipoxygenase. Except for LA and OA, all other ligands bind to a shallow cleft defined by Gln^65^-Lys^71^, Gly^105^-Leu^111^ and Leu^127^-Lys^140^ on the surface of one monomer. On the other hand, the LA and OA molecules attach with high affinity to narrow cavity delimited by the Val^107^-Asp^113^ and Leu^127^-Gln^139^ (Figure 2c). Given the intrinsic instability of the enzyme [29], even though the tested ligands do not bind near the active site, their attachment to specific cavities might result in local conformational changes, which can further influence the conformation of the active site. Notwithstanding, when attempting to explain the experimental results on inhibitory effect on metabolic syndrome-associated enzymes, although at rather low concentration, the presence of other phytochemicals originating from SBP should not be neglected. 

### 2.4. Evaluation of Physical Characteristics of the Microcapsule Powders

The flow properties of the powders are very important from technological perspective, especially when the powders are used as final forms [31]. The physical parameters of the investigated powders are presented in Table 4. 

The highest BD was recorded for V1 and the lowest for V4, indicating that glycation treatment exerted a significant effect on the flow properties of the powders. Regarding the TD, the powder obtained with WPI, and pectin (V2) presented the lowest value, whereas V1 and V3 had similar TD values. Although the differences between samples based on CI and HR values are significant (*p* < 0.01), all the powders exhibited poor flowing properties. Our results are in line with those reported by Bordon et al. [32]. The authors reported that microencapsulated oils are characterized by poor flowability, which was associated with moisture content, non-encapsulated oil and electrostatic interaction between particles. Other studies pointed out that powder morphology has an impact on their flowing properties—powders with irregular shape being characterized as possessing reduced flowability [33].

Moisture and a_w_ are two of the most known parameters used for evaluation of storage stability, higher values than 10% for moisture and 0.3 for a_w_ promoting microbial and chemical changes of the powders [34]. The moisture content of the powders ranged between 6% and 9%, whereas a_w_ ranged between 0.084 ± 0.03 and 0.241 ± 0.003, suggesting that all the tested powders are stable to storage. 

In case of solubility, significant differences (*p* < 0.05) were noticed between non-heated samples and glycated samples. For non-heated samples (V1, V2, V3), the results (Table 4) showed that powder containing inulin (V1) was the most soluble, whereas powder containing pectin (V2) presented the lowest solubility (34 ± 1%). Glycation of WPI with inulin decreased powder solubility by about 53%, whereas glycation of WPI with lactose increased powder solubility by about 5%, indicating that solubility was dependent by the type of carbohydrate used in glycation of WPI.

### 2.5. Structural and Morphological Analysis of the Powders

**Figure 3:**
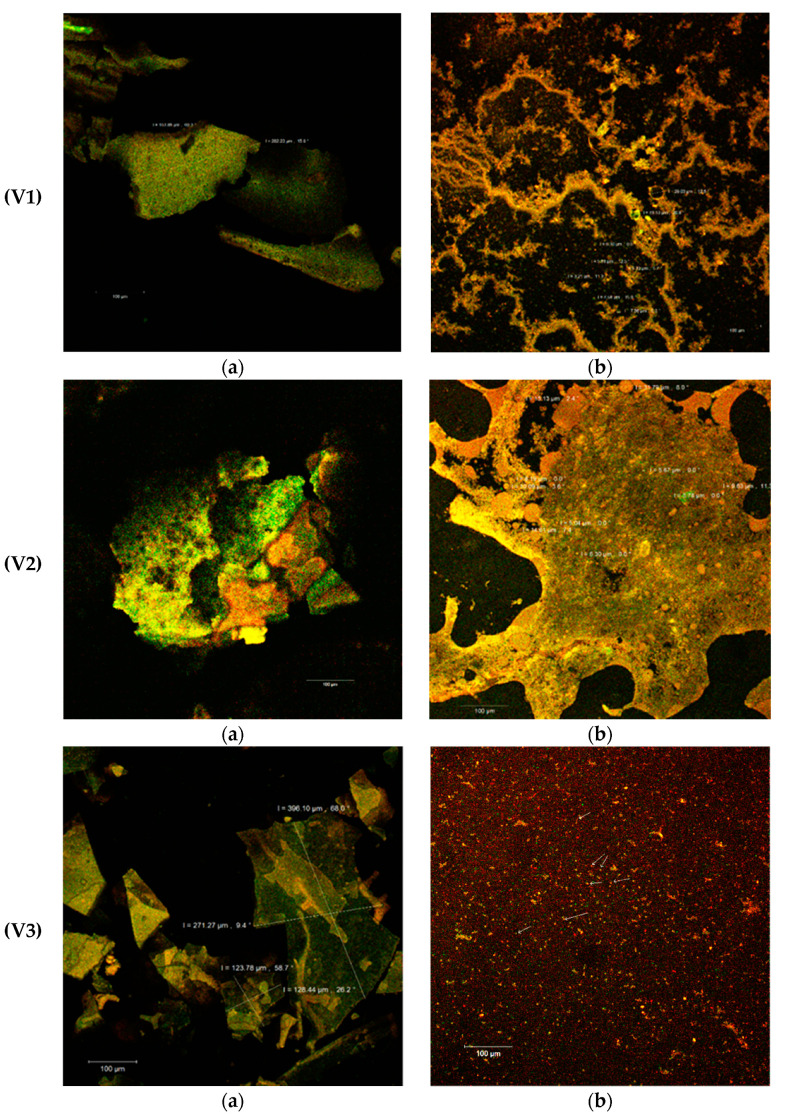

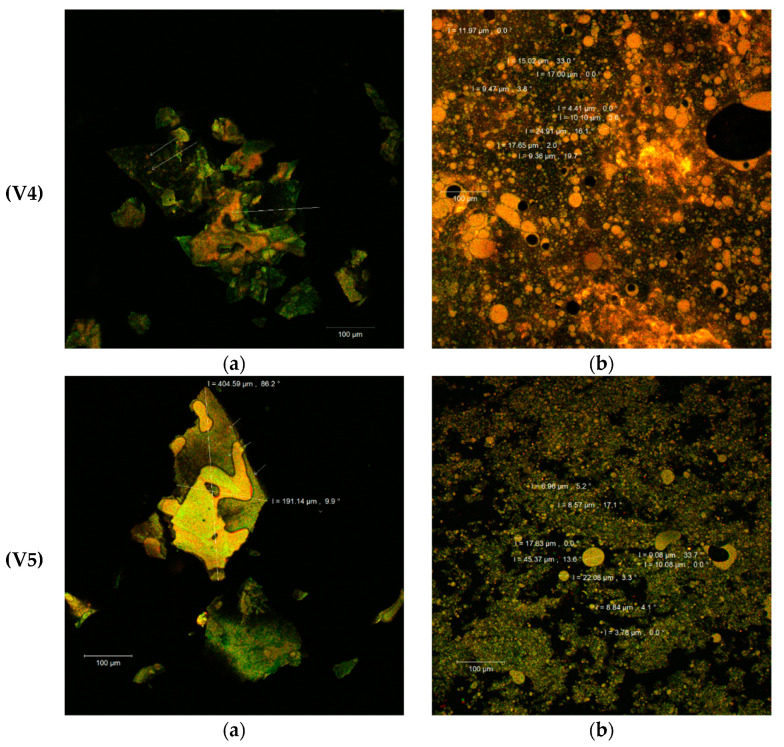
Microscopic details of the microencapsulated powders (V1–V5) in the native variants (**a**) and fluorescently labeled (**b**) by laser scanning with a Zeiss confocal system (LSM 710).

The confocal analysis of the experimental variants completes the performed biochemical analyzes and provides some additional information regarding the morphology of the powders obtained from SBP through complex microencapsulation techniques: emulsion, coacervation and freeze-drying, using several biopolymer matrix combinations (WPI-I/P/L). The samples without the fluorescence labeling displayed the formation of thin, fragile biofilms by incorporating SBP oleoresins into the complex matrix. The size of the scaly formations appeared to be between 164–282 µm (V1) and 191–405 µm (V5). The V5 sample was distinguished as having the largest thickness of the biofilm. In the structure of these films, several microvesicles could be observed due to the autofluorescence of the biologically active compounds from SBP (see the white arrows in Figure 3V2a,V4a,V5a).

As known, carotenoids, that predominate in the SBP, have emission wavelengths around 630, 685 and 750 nm, depending on the combination of pigments in the plant source and on the interactions established with the other biologically active compounds (fatty acids, sterols, etc.) [35]. The Congo Red staining of the samples (Figure 3b) demonstrated the microencapsulation of the SBP biocompounds in the form of spherosomes, with variable aggregation behavior. The spherosomes are finer in the V1 (3–29 µm diameter) and V3 (<2 µm diameter) variants and have a reduced clustering capacity (Figure 3V1b,V3b). The microvesicles obtained through a complex encapsulation technique in the V4 and V5 experimental variants were more uniform and aggregated in the form of coacervates into a three-dimensional network. Similar results were obtained by Neagu et al. [36] who used a cross-linking treatment with transglutaminase on WPI and casein as matrix. The heat treatment at 90 °C significantly improved the microscopic appearance of the powders probably due to the exposure of the binding sites that allowed the interaction of the polymer matrix components (WPI and I or L), so as to entrap by coacervation and freeze drying the spherosomes with the SB oleoresins. More than 80% of the microcapsules presented a diameter <10 µm for the V5 variant (Figure 3V5b), which makes it more efficient, more stable and, thereby, more functional.

## 3. Materials and Methods

### 3.1. Materials

SBP were obtained from the local juice producer (S.C. Bio-Hipporham SRL, Urzica village, Olt County, Romania), dried at 35 °C in a laboratory-fluidized bed-dryer FT-31 model (Armfield, UK) and ground by a hand-mill with 180W electric power (Bosch, Germany). WPI (protein content of 95%) was purchased from Fonterra (New Zeeland). Inulin (from chicory, ≥90%), pectin from apple (50–75% esterification) and lactose anhydrous were purchased from Merck KGaA (Darmstadt, Germany). Lipoxidase from Glycine max (soybean) type I-B, 50,000 units/mg protein, pancreatin lipase (111.5 units/mg protein), α-amylase from porcine pancreas (type I-A, 700–1400 units/mg protein), sodium phosphate buffer solution (PBS), linoleic acid (≥99%), p-nitrophenyl palmitate, Arabic gum, Triton X-100, starch solution, dinitrosalicylic acid (DNS), were purchased from Sigma Aldrich (Steinheim am Albuch, Germany). All reagents and solvents were of analytical and HPLC grade.

### 3.2. SFE-CO_2_ Extraction

The SFE-CO_2_ extractions of oleosome from SBP was performed as described by Mihalcea et al. [37], using a pilot plant extraction equipment (Natex Prozesstechnologie GesmbH, Ternitz, Austria). In brief, about 0.4 kg of SBP (91.34 ± 0.35% dry matter) was loaded into the extraction vessel. The air in the system was removed by flushing the system with CO_2_ and the extractor was pressurized with CO_2_ (99.999% purity supplied by Messer S.A., Bucuresti, Romania) using a high-pressure CO_2_ pump. The extraction was performed for 105 min at 35 °C and 45 MPa. The CO_2_ flow was monitored with a Coriolis mass flow meter and generated by the data sheets from ABB software (ABB, Mannheim, Germany). The average CO_2_ mass flow for the extraction batches was 21.23 ± 0.61 kg/h. In order to produce fractions with different composition, the pressure in the first separator S40 was maintained constant at 15 MPa, while in the second separator (S45) decompression up to recirculation pressure of 5 MPa was set [38]. After extraction, the obtained fractions corresponding to the separators were mixed together and kept into dark bottles under refrigeration conditions (2–4 °C) until further use in the experiments. 

### 3.3. Microencapsulation of SBP Oleoresins

To prepare microcapsules, the initial biopolymeric combination was formed by hydrating WPI in deionized water to prepare 3% *w*/*v* solution. Then, equal weight of inulin (I, variant 1), pectin (P, variant 2), lactose (L, variant 3) was dissolved in the same solution to end up with total solid content 4.5% *w*/*v* (WPI:I/P/L 2:1, *w*/*w*). The biopolymeric solutions were allowed to mix on a magnetic stirrer until complete hydration at 450 rpm and 40 °C for 4 h. Variant 4 and 5 containing WPI:I (V4) and WPI:L (V5) were subjected to thermal treatment at 90 °C/3 h using a heating block (Stuart SBH200D, UK). About 7 mL of each of the solutions were filled in glass tubes, sealed and heated. After heating, the tubes were cooled in ice water. Further, about 3 g of SBP oleoresins was added into the aqueous solutions and homogenized using an Ultra Turrax mixer (IKA T18 basic) at 5000× *g* for 10 min to form a coarse emulsion. The complex coarcevation of the reaction mixture was promoted by lowering the pH to 3.75 with 1N HCl solution at 40 ± 1 °C, under constant mechanical stirring at 600 rpm. The reaction mixtures were then allowed to cool in the ice bath and stored at 4–6 °C overnight, to promote decantation. The coacervates were freeze-dried (CHRIST Alpha 1-4 LD plus, Osterode am Harz Germany) at −42 °C under a pressure of 10 Pa for 48 h. Afterwards, the powders were collected and packed in metallized bags, and kept in the refrigerator at 4 °C until further analysis.

### 3.4. Microencapsulation Efficiency

For microencapsulation efficiency of phytochemicals, the method described by Gheonea (Dima) et al. [39] was applied, by estimating the total (TC) and surface (SC) carotenoids. In brief, for TC estimation, about 50 mg of powders were dissolved in 6 mL of 10% NaCl:methanol (ratio of 1:1). The mixtures were sonicated for 30 min at 40 °C to break the microcapsules, followed by addition of a volume of 30 mL *n*-hexane:acetone mixture (1:1) and centrifugation at 6000× *g* for 10 min. Absorption was read in a spectrophotometer in the visible wavelength at selected wavelengths (450 nm, 470 nm and 503 nm). 

The SC were measured by dissolving about 50 mg of powders in 10 mL of 1:1 (*v*/*v*) *n*-hexane:acetone mixture, followed by a short agitation with a vortex, and centrifugation at 1500× *g* for 3 min. The optical density of the upper organic fraction was measured as mentioned above. Encapsulation efficiency was calculated using Equation (1):

(1) $$Microencapsulation\;efficiency\;\left(\%\right)=\frac{TC-SC}{TC}\times 100$$

### 3.5. Global Phytochemicals Profiling and Antioxidant Activity

The extract and powders were characterized in terms of total carotenoids, β-carotene, lycopene and antioxidant activity using spectrophotometric methods. About 5 mg of concentrated extracts were dissolved in 10 mL of a mixture of *n*-hexane: acetone (ratio of 3:1), in a volumetric flask. For powders, the supernatants resulted from the extraction of total carotenoids, as mentioned above, were used for phytochemicals and antioxidant assays. Different wavelengths were used for carotenoids evaluation, such as 450 nm for total carotenoid content, 470 nm for β-carotene and 503 nm for lycopene. The carotenoids amount was expressed as mg/g dry weight (DW) according to the Equation (2):

(2) Carotenoids mgg=A×MW×Df/Ma×L

with: *A*—absorbance at 470 nm, 450 nm, 503 nm; *M_w_*: molecular weight for lycopene and β-carotene (536.873 and 536.87 g·mol^−1^, respectively), *D_f_*—sample dilution rate, *M_a_*—molar absorptivity in n-hexane (3450 L mol^−1^ cm^−1^ for lycopene, 2592 L mol^−1^ cm^−1^ for total carotenoids, and 2500 L mol^−1^ cm^−1^ for β-carotene), and L—cell diameter of the spectrophotometer (1 cm).

The radical scavenging ability was evaluated using TEAC (Trolox Equivalent Antioxidant Capacity) by using the 2,2-azinobis-3-ethyl-benzothialzoline-6-sulfonic acid (ABTS^∙+^) radical cation decoloring reaction, as described by Mihalcea et al. [37]. In brief, volumes of 0.15 mL of extracts dissolved in *n*-hexane:acetone mixture and supernatants resulted from the extraction of total carotenoids from powders were added to a volume of 2.85 mL of the ABTS^∙+^ solution and allowed to react for 2 h in the dark. The absorbance of the mixture was measured at 734 nm. The ABTS^∙+^ antioxidant activity of the extracts was expressed as mMol TEAC/g DW. 

### 3.6. Fatty Acids, Phytosterols and Tocopherols Content of the Extract and Powders

Fatty acids, phytosterols and tocopherols contents of the extract and powders were determined as described by Mihalcea et al. [37]. In brief, the fatty acids contents were converted in their respective methyl esters (FAMEs) using the methanolic boron trifluoride (BF_3_)-catalyzed esterification procedure, using a gas-chromatographic system coupled with mass spectrometer (GC-MS) (Perkin Elmer Clarus 680/SQ 8T, Perkin Elmer, Norwalk, CT, USA) equipped with an Elite-WAX capillary column (30 m × 0.25 mm i.d., 0.25 µm film thickness (Perkin Elmer, Norwalk, CT, USA), using helium as carrier gas (flow rate of 1.5 mL/min). The quantification of FAMEs present in the extracts was performed in selected ion recording mode (SIR), using a 5-points calibration curve prepared from 37 component FAME Mix (Supelco, Sigma-Aldrich, Darmstadt, Germany). The phytosterols and tocopherols profile of SBP extract and powders was determined via GC-MS using the extracts and powders dissolved in *n*-hexane as mentioned above. 

### 3.7. In Vitro Digestion

In order to evaluate the carotenoids’ bio-accessibility from the powders, the three static in vitro digestion model was used, including short oral, gastric and intestinal simulated stages, as described by Szabo et al. [20]. Given the presence of carbohydrates in the microencapsulated samples, it was decided to evaluate the resistance time of carotenoids in the oral cavity. In brief, 1 g of each powder was allowed to hydrate in 6 mL of simulated saliva (comprising 0.012 g NaCl, 0.015 g KCl, 0.21 g NaHCO_3_, 0.04 g urea, 0.1 g mucin, 0.2 g α-amylase, and water to make up to 100 mL, with the pH adjusted to 6.8 using NaOH or HCl) to each sample (as stated above) and mixing in a 50 mL flask. These mixtures were adjusted to pH 6.8 and then shaken continuously at 95 rpm in a 37 °C temperature-controlled incubator (Incubated Shaker SI-300R JEIO TECH, Vilnius, Lithuania) for 10 min, and then added to 8 mL of simulated gastric juice (comprising 0.2 g NaCl, 0.7 mL HCl, 0.32 g pepsin, and water to make up to 100 mL, with the pH adjusted to 1.2 using 1.0 M HCl to the flasks). The mixtures were allowed to react for 2 h at 37 °C and 95 rpm in an incubator shaker. After 2 h of reaction in the gastric environment, the mixtures were added to 8 mL in simulated intestinal juice (3 mL of pancreatic lipase solution (4.8 mg/mL), 4 mL of bile extract solution (5 mg/mL), and 1 mL of calcium chloride solution (750 mM) and the mixtures were incubated at 37 °C for 2 h, under rotation at 95 rpm in a temperature-controlled incubator (Incubated Shaker SI-300R JEIO TECH, Vilnius, Lithuania). At every digestion stage, a volume of 1 mL of each sample was immediately centrifuged at 14,000× *g* for 10 min (Ultracentrifuge with cooling, HETTICH Universal 320 R, Germany), in order to minimize the enzyme activity and to remove the undigested material. Carotenoids bio-accessibility was calculated as the ratio between carotenoid concentration in the supernatant of the intestinal fraction and its initial concentration powders (Equation (3)).

(3) $$Bioaccesibility\;\left(\%\right)=\frac{Carotenoid\;concentration_{supernatant}}{Carotenoid\;concentration_{powder}}\times 100$$

### 3.8. Inhibitory Effect on Metabolic Syndrome-Associated Enzymes

The inhibitory effect of the powders on metabolic syndrome-associated enzymes was tested on α-amylase, lipase and lipoxygenase, as described by Condurache et al. [26]. In brief, 50 µL of 1, 0.5, and 0.1 mg/mL of the powders diluted in ultrapure water was added to 50 µL of enzymes solution (1 mg/mL in 0.1 M PBS of pH = 9.0 for lipoxygenase, pH = 8.0 for lipase and pH = 6.9 for α-amylase). The mixtures were incubated for 5 min at room temperature, followed by the addition of selected substrate, as follow: 50 µL of 0.05 mM linoleic acid (in 0.1 M PBS, pH = 9.0), 50 µL of 0.01 M p-nitrophenyl palmitate, Arabic gum, and Triton x-100, and 100 µL of soluble starch 1% solution dissolved in distilled water and previously boiled for 5 min. The mixtures were incubated for 20 min at 37 °C. For α-amylase, the protocol involved the addition of 200 µL of DNS, followed by heating at 100 °C for 5 min in a thermostatic water bath (Digibath-2 BAD 4, Raypa Trade, Barcelona, Spain). After incubation, each sample was diluted with 0.1 M PBS at the appropriate pH, followed by the absorbance reading at the selected wavelength (243 nm for lipoxygenase, 400 nm for lipase and 540 nm for α-amylase), using a double-beam UV-VIS spectrophotometer with data analysis software (Jenway, UK). Quercitin, orlistat and metformin hydrochloride were used as the standard inhibitors for selected enzymes. The enzyme activity inhibition was calculated using the following Equation (4):

(4) $$\%Inhibition=\frac{\left(A_{0}-A_{S}\right)}{A_{0}}\times 100$$

where *A*_0_ is the absorbance of the control sample without powder; *A_s_* is the absorbance of the sample with the powder. The inhibitory effect of the powders was expressed as IC50 (µg/mL).

### 3.9. Molecular Modelling Investigation on Phytochemical Binding to the Enzymes

The PatchDock algorithm [40], which performs rigid docking based on the molecular shape complementarity was used to probe the potential interference of the main phytochemicals from SBP oleoresins, with the activity of the metabolic syndrome-associated enzymes. In order to perform the docking tests, the 3D crystallography models of the α-amylase (6Z8L.pdb, [27]), lipase (1N8S.pdb, [41]), and lipoxygenase (3O8Y.pdb, [29]) were taken from the RCSB Protein Data Bank and after refinement were used as receptors. The major phytochemicals identified by means of GC-MS in the SBP powders were used as ligands in the molecular docking procedure. The molecular models of β-caroten (BCR), palmitic acid (PA), oleic acid (OA), linoleic acid (LA), β-sitosterol (BST) and β-tocopherol (BTF) were prepared and optimized using Hyperchem 8.0 software (Hypercube Inc., Waterloo, ON, Canada). The best fits ranked on the bases of the interaction energy values for each of the eighteen protein-ligand complexes were further carefully checked for identifying any atomic level contact, which might help in explaining the mechanisms involved in enzymes inhibition.

## 3.10. Evaluation of Physical Characteristics of the Microcapsule Powders

The flow properties of the powders were expressed in terms of bulk density (BD, kg/m^3^), tapped density (TD, kg/m^3^), Carr Index (CI) and Hausner ratio (HR), as previously reported by Dumitrașcu et al. [34]. Water activity (aw) was determined using a water activity analyzer (GBX Fast-lab, Dublin, Ireland); water content was evaluated using a moisture analyzer (AND MF-50, Tokyo, Japan). Solubility was evaluated as follows: 200 mg powder sample was mixed with 10 mL of distilled water and vigorously mixed for 30 min. Then, the solution was centrifuged at 6000× *g*, for 10 min. A volume of 9 mL of supernatant was oven-dried at 105 °C until constant weight. The solubility (%) was expressed as the difference between the supernatant before drying and after drying. 

## 3.11. Structural and Morphological Analysis of the Powders

The confocal laser scanning microscopy (CLSM) analysis was performed to determine the structural appearance and characteristics of the powders. The images were captured with a LSM710 Zeiss Confocal Laser Scanning System, which is a system equipped with several types of lasers such as a diode laser (405 nm), Ar-laser (458 nm, 488 nm and 514 nm), DPSS laser (diode pumped solid state—561 nm) and HeNe-laser (633 nm). The powders were observed with a 20 × apochromatic objective, zoom 1. The obtained 3D images were rendered and processed with the ZEN 2012 SP1 software (Black Edition).

## 3.12. Statistical Analysis

The results are expressed in terms of average followed by standard deviation. The statistical evaluation was performed using Minitab 19 software. First, the data were checked for normality (Ryan–Joiner test) and homoscedasticity condition. The differences between samples were analyzed using ANOVA method, and the post hoc analysis was performed based on Tukey or Games Howell method (when the homoscedasticity condition was not confirmed).

## 4. Conclusions

Given the actual trend in developing innovative technological approaches for the effective extraction of bioactives for food, nutraceutical, pharmaceutical and cosmetics applications, sea buckthorn pomace was used as a source of oleoresin as an alternative to revalorize the low-cost renewable resources, concomitant with reducing wastes, and positive economic and environmental impacts. The supercritical carbon dioxide extraction was applied, whereas the resulted oleoresins were microencapsulated in different combinations of matrices, based on whey proteins isolate. Therefore, inulin, pectin and lactose were used as adjuvants for microencapsulation of oleoresins, with and without glycation. All variants showed satisfactory phytochemical content, in term of carotenoids, fatty acids, tocopherols, phytosterols, and antioxidant activity, with a particular emphasis on the palmitoleic acid content in the samples obtained on whey protein isolate: inulin conjugation basis. All the samples demonstrated an inhibitory effect on relevant metabolic enzymes, excepting for the variant with pectin, which showed no effect on α-amylase. The microencapsulation had a significant effect on the bio-accessibility of carotenoids in all variants, when compared with the free extract. From a structural point of view, the nonconjugated samples displayed finer particles, with aggregational behavior, whereas conjugated forms showed more uniform and aggregated microparticles, into a three-dimensional network. From the variants tested, it can be concluded that the encapsulation of sea buckthorn bioactive extract in the conjugated complex (whey protein isolate and lactose/inulin) may be a promising technique to deliver powders with enhanced quality characteristics and functionality. Future studies should consider the stability of bioactives during storage, whereas the behaviour of the powders should be tested in different applications.

## Figures and Tables

**Table 1:** Comparative analysis of the phytochemical profile of the SBP oleoresins and microencapsulated powders.

Phytochemicals	Extract	V1	V2	V3	V4	V5
Carotenoids (mg/g DW)						
Total carotenoids	510 ± 8	143.0 ± 0.3 ^c^	120.0 ± 0.6 ^d^	179 ± 2 ^b^	199.0 ± 0.4 ^a^	178 ± 1 ^b^
β-carotene	432 ± 6	122.0 ± 0.3 ^c^	101.0 ± 0.2 ^d^	152.0 ± 0.8 ^b^	168 ± 2 ^a^	151.0 ± 0.4 ^b^
Lycopene	88 ± 2	27.0 ± 0.2 ^c^	23.0 ± 0.4 ^d^	31.0 ± 0.4 ^b^	34.0 ± 0.4 ^a^	35.1 ± 0.8 ^a^
Fatty acids (mg/g)						
Myristic acid (C14:0)	3.1 ± 0.2	2.1 ± 0.1	2.1 ± 0.1	1.5 ± 0.1	2.1 ± 0.1	2.1 ± 0.1
Pentadecanoic acid (C15:0)	1.5 ± 0.1	0.8 ± 0.1	1.01 ± 0.03	1.0 ± 0.1	1.0 ± 0.1	1.01 ± 0.01
Palmitic acid (C16:0)	92 ± 2	16.2 ± 1.5	19 ± 1	12 ± 1	23 ± 1	28.1 ± 0.5
Palmitoleic acid (C16:1)	58 ± 1	12 ± 1	13 ± 1	8 ± 1	16 ± 3	20.1 ± 0.4
Stearic acid (C18:0)	15.1 ± 0.7	4 ± 1	4.0 ± 0.5	4 ± 1	5 ± 1	6 ± 1
Oleic acid (C18:1)	77 ± 1	17 ± 2	20 ± 2	12 ± 1	23 ± 2	30 ± 2
Linoleic acid (C18:2)	158 ± 1	16 ± 1	34 ± 2	21 ± 2	22 ± 2	46 ± 1
γ-Linolenic acid (C18:3)	115.0 ± 2.3	18 ± 2	22 ± 2	14 ± 1	14 ± 1	31 ± 2
Gondoic acid (C20:1)	1.5 ± 0.1	0.40 ± 0.01	0.4 ± 0.1	0.3 ± 0.1	0.5 ± 0.1	0.61 ± 0.03
cis-11,14-Eicosadienoic acid (C20:2)	1.1 ± 0.1	0.8 ± 0.1	0.8 ± 0.1	0.71 ± 0.05	0.8 ± 0.1	0.8 ± 0.1
cis-5,8,11,14,17-Eicosapentanoic acid (C20:5)	1.01 ± 0.06	0.9 ± 0.1	0.9 ± 0.1	0.9 ± 0.1	0.9 ± 0.1	0.9 ± 0.1
Tocopherols (µg/g)						
α-tocopherol	1040 ± 22	591 ± 12	80 ± 2	160 ± 5	161 ± 5	540 ± 11
β-tocopherol	1290 ± 16	101 ± 2	93 ± 3	57 ± 3	107 ± 2	163 ± 4
γ-tocopherol	20 ± 1	9 ± 1	9 ± 1	4 ± 1	11 ± 1	14 ± 1
Phytosterols (% from total peak)						
Campesterol	2.1 ± 0.2	2.1 ± 0.2	2.1 ± 0.3	2.0 ± 0.1	2.0 ± 0.1	2.1 ± 0.4
β-Sitosterol	96 ± 2	95 ± 3	95 ± 3	96 ± 4	95 ± 3	95 ± 3
β-Amyrin	1.0 ± 0.1	1.1 ± 0.1	1.1 ± 0.1	1.0 ± 0.1	1.1 ± 0.1	1.0 ± 0.1
α-Amyrin	1.1 ± 0.1	2.0 ± 0.3	2.0 ± 0.1	2.0 ± 0.1	2.1 ± 0.1	2.1 ± 0.1
**Antioxidant activity (mMol TEAC/g DW)**						
	32.2 ± 0.2	12 ± 1 ^ab^	11.1 ± 0.1 ^b^	11.2 ± 0.1 ^b^	14 ± 1 ^ab^	14.0 ± 0.3 ^a^

For carotenoids content, means that for the same row do not share the same superscript letter (a, b, c, d), are statistically different at *p* < 0.001 based on ANOVA and Tukey method. For antioxidant activity, means that do not share the same superscript letter (a, b) are significant at *p* < 0.001 based on ANOVA and Games–Howell method and 95% confidence.

**Table 2:** Encapsulation efficiency of the selected phytochemicals in microencapsulated variants.

Microencapsulation Efficiency (%)	Microencapsulated Variants				
	V1	V2	V3	V4	V5
Total carotenoids	88.5 ± 0.1 ^d^	91.5 ± 0.1 ^a^	87.1 ± 0.1 ^e^	89.3 ± 0.1 ^b^	88.5 ± 0.1 ^c^
β-carotene	87.1 ± 0.2 ^cd^	91 ± 1 ^a^	87.1 ± 0.1 ^d^	89.3 ± 0.1 ^b^	88.4 ± 0.2 ^c^
Lycopene	82.2 ± 0.5 ^b^	82 ± 1 ^b^	81.2 ± 0.2 ^c^	86.5 ± 0.7 ^a^	83.1 ± 0.7 ^b^

Means that for the same row do not share the same superscript letter (a, b, c, d, e) are statistically significant at *p* < 0.001 based on ANOVA and Tukey method.

**Table 3:** The enzyme inhibition results (IC50 values; µg/mL) of the microencapsulated powders.

Enzyme	IC_50_ (µg/mL)				
	V1	V2	V3	V4	V5
α-amylase	19.1 ± 0.4 ^b^	0	25 ± 2 ^ab^	28.0 ± 0.2 ^a^	29.0 ± 0.3 ^a^
Lipase	31.1 ± 0.3 ^bc^	30.1 ± 0.2 ^bc^	30 ± 1 ^c^	31 ± 1 ^ab^	32.0 ± 0.3 ^a^
Lipoxygenase	33.1 ± 0.1 ^b^	36.1 ± 0.3 ^a^	26 ± 1 ^c^	37 ± 1 ^a^	23.0 ± 0.1 ^c^

For α-amylase and lipoxygenase, means that do not share the same superscript letter (a, b, c) indicate significant differences between variants at *p* < 0.001 based on ANOVA and Games–Howell method and 95% confidence. For lipase activity, means that do not share the same superscript letter (a, b, c) indicate significant differences between variants at *p* < 0.001 based on ANOVA and Tukey method.

**Table 4:** Physical parameters of the microencapsulated powders.

Parameter	Microencapsulated Variants				
	V1	V2	V3	V4	V5
BD (kg/m^3^)	118 ± 1 ^a^	83 ± 2 ^c^	91 ± 1 ^b^	62 ± 7 ^d^	71 ± 5 ^d^
TD (kg/m^3^)	193 ± 8 ^a^	132 ± 2 ^d^	196 ± 8 ^a^	150 ± 20 ^c^	177 ± 11 ^b^
CI	38 ± 3 ^c^	32 ± 1 ^d^	54 ± 1 ^b^	58 ± 2 ^a^	60.1 ± 0.5 ^a^
HR	2.0 ± 0.1 ^c^	2.01 ± 0.02 ^c^	2.0 ± 0.1 ^b^	2.0 ± 0.1 ^a^	2.01 ± 0.02 ^a^
a_w_	0.118 ± 0.002 ^b^	0.084 ± 0.03 ^e^	0.104 ± 0.009 ^c^	0.241 ± 0.003 ^a^	0.088 ± 0.001 ^d^
Moisture content (%)	7 ± 1 ^b^	7.0 ± 0.5 ^b^	6.0 ± 0.3 ^c^	8 ± 1 ^a^	9 ± 1 ^a^
Solubility (%)	71 ± 6 ^a^	34 ± 1 ^d^	60 ± 0.00 ^c^	33.00 ± 0.00 ^d^	66 ± 4 ^b^

Mean values that for same parameter do not share the same superscript letter (a, b, c, d, e) are significant at *p* < 0.01 based on ANOVA and post hoc Tukey test.

## Data Availability

Data is contained within the article.
